# Label-free quantitative proteomics reveals aberrant expression levels of LRG, C9, FN, A1AT and AGP1 in the plasma of patients with colorectal cancer

**DOI:** 10.1186/s12014-023-09407-y

**Published:** 2023-04-06

**Authors:** Chris Verathamjamras, Juthamard Chantaraamporn, Thiwaree Sornprachum, Photsathorn Mutapat, Daranee Chokchaichamnankit, Kanokwan Mingkwan, Virat Luevisadpibul, Chantragan Srisomsap, Somchai Chutipongtanate, Jisnuson Svasti, Voraratt Champattanachai

**Affiliations:** 1 Laboratory of Biochemistry, Chulabhorn Research Institute, Bangkok, Thailand; 2 Division of Surgery, Sapphasitthiprasong Hospital, Ubon Ratchathani, Thailand; 3 Division of Information and Technology, Ubonrak Thonburi Hospital, Ubon Ratchathani, Thailand; 4 Pediatric Translational Research Unit, Department of Pediatrics, Faculty of Medicine Ramathibodi Hospital, Mahidol University, Bangkok, Thailand; 5 Department of Environmental and Public Health Sciences, University of Cincinnati College of Medicine, Cincinnati, OH USA; 6 Applied Biological Science Program, Chulabhorn Graduate Institute, Chulabhorn Royal Academy, Bangkok, Thailand

**Keywords:** Affinity chromatography, blood-based biomarkers, colorectal cancer, label-free quantitative proteomics, MARS-14

## Abstract

**Background:**

Colorectal cancer (CRC) is one of the major causes of cancer-related death worldwide. Although commercial biomarkers of CRC are currently available, they are still lacking in terms of sensitivity and specificity; thus, searching for reliable blood-based biomarkers are important for the primary screening of CRC.

**Methods:**

Plasma samples of patients with non-metastatic (NM) and metastatic (M) CRC and healthy controls were fractionated using MARS-14 immunoaffinity chromatography. The flow-through and elute fractions representing low- and high-abundant proteins, respectively, were analyzed by label-free quantitative proteomics mass spectrometry. The functional analysis of the proteins with greater than 1.5-fold differential expression level between the CRC and the healthy control groups were analyzed for their biological processes and molecular functions. In addition, the levels of plasma proteins showing large alterations in CRC patients were confirmed by immunoblotting using two independent cohorts. Moreover, receiver operating characteristic (ROC) curve analysis was performed for individual and combinations of biomarker candidates so as to evaluate the diagnostic performance of biomarker candidates.

**Results:**

From 163 refined identifications, five proteins were up-regulated and two proteins were down-regulated in NM-CRC while eight proteins were up-regulated and three proteins were down-regulated in M-CRC, respectively. Altered plasma proteins in NM-CRC were mainly involved in complement activation, while those in M-CRC were clustered in acute-phase response, complement activation, and inflammatory response. Results from the study- and validation-cohorts indicate that the levels of leucine-rich alpha-2-glycoprotein-1(LRG), complement component C9 (C9), alpha-1-acid glycoprotein 1 (AGP1), and alpha-1-antitrypsin (A1AT) were statistically increased, while fibronectin (FN) level was statistically decreased in CRC patients compared to healthy controls, with most alterations found in a metastatic stage-dependent manner. ROC analysis revealed that FN exhibited the best diagnostic performance to discriminate CRC patients and healthy controls while AGP1 showed the best discrimination between the disease stages in both cohorts. The combined biomarker candidates, FN + A1AT + AGP1, exhibited perfect discriminatory power to discriminate between the CRC population and healthy controls whereas LRG + A1AT + AGP1 was likely to be the best panel to discriminate the metastatic stages in both cohorts.

**Conclusions:**

This study identified and quantified distinct plasma proteome profiles of CRC patients. Selected CRC biomarker candidates including FN, LRG, C9, A1AT, and AGP1 may be further applied for screening larger cohorts including disease groups from other types of cancer or other diseases.

**Supplementary Information:**

The online version contains supplementary material available at 10.1186/s12014-023-09407-y.

## Background

Colorectal cancer (CRC) is one of the most common cancers worldwide. Global cancer statistics of 2020 indicate that it ranks as third in mortality (10.0%) and as second in morbidity (9.4%) of all cancers [1]. Generally, the symptoms of patients with CRC may not be obvious until the disease has progressed to advanced stages. Currently, the gold standard for CRC diagnosis is to examine the presence of malignant cells in the biopsy tissues using colonoscopy; however, it is costly and invasive for the patients. Until now, carcinoembryonic antigen (CEA) and carbohydrate antigen 19 − 9 (CA19-9) are two common blood-based biomarkers for monitoring CRC patients [2]. However, increasing level of CEA in the blood is not specific for patients with CRC, but can also be found in other diseases such as inflammatory bowel disease and other malignancies, while CA19-9 is less sensitive and less specific for CRC [2]. Nevertheless, the search for specific biomarkers from liquid biopsy specimens such as plasma and serum is still an important non-invasive approach for primary screening of CRC.

Mass spectrometry (MS)-based proteomics has become a powerful tool for studies aimed at biomarker discovery, as well as for plasma proteome research [3]. However, finding biomarkers in plasma samples is challenging because plasma contains numerous proteins that may vary in concentration among individuals. In addition, proteins of interest may be overshadowed by certain high-abundant proteins such as albumin and immunoglobulins. Several immunoaffinity separations coupled with liquid chromatography-tandem mass spectrometry (LC-MS/MS) have been developed to search for low-abundance proteins which may reflect the pathophysiological conditions of patients [4]. Among them, the Human 14 Multiple Affinity Removal System (MARS-14) has been wildly used for cancer biomarker research such as for cervical cancer [5], colorectal cancer [6], gastric cancer [7], biliary tract cancer [8] and lung cancer [9]. Although the combination of LC-MS/MS-based proteomics and immunoaffinity separations is an effective technique for discovery of potential biomarkers, data interpretation and selection of candidate proteins can be still challenging.

The purpose of this study is to identify biomarker candidates for CRC screening. Plasma samples from healthy controls and patients with CRC in non-metastatic and metastatic groups were investigated using MARS-14 immunoaffinity chromatography and LC-MS/MS. The low- and high-abundance proteins were separated and both fractions were identified and quantified using label-free quantitative proteomic analysis. MS data interpretation and peptide selections were intensively performed and combined in order to obtain reliable peptides/proteins for comparison among these groups. In addition, the candidate proteins were confirmed by immunoblotting in two independent cohorts. Furthermore, the diagnostic performance of single and combined candidate proteins was assessed using receiver operating characteristic (ROC) curve analysis.

## Methods

### Patients and specimens

EDTA-plasma samples were collected as left-over specimens at Sappasitthiprasong Hospital in Ubon Ratchathani, Thailand. The samples were frozen, transferred and thawed to make aliquots before being kept at -80˚C until use. The study-cohort (n = 30) included 20 CRC patients (10 metastatic-staged patients and 10 non-metastatic-staged patients) and 10 healthy controls. The validation-cohort (n = 45) included 30 CRC patients (16 metastatic-staged patients and 14 non-metastatic-staged patients) and 15 healthy controls. Individuals who presented to the hospital for annual check-up and had no history of underlying illness were considered healthy controls. The characteristics of all samples are summarized in Table 1 and the supplementary data, Table S1. This study was approved by the local ethics committee of Faculty of Medicine Ramathibodi Hospital, Mahidol University and Sappasitthiprasong Hospital (protocol ID 03-58-68; approved on May 8, 2015; last amended on May 4, 2018). Written informed consent was waived due to the use of discarded de-identified specimens.

**Table 1:** Characteristics of CRC patients and healthy controls used in the study- and validation-cohorts.

Descriptions	Study-Cohort			Validation-Cohort		
	Healthy Controls	Non-Metastatic CRC	Metastatic CRC	Healthy Controls	Non-Metastatic CRC	Metastatic CRC
Numbers	10	10	10	15	14	16
Gender						
Female	4	4	4	15	5	11
Male	6	6	6	0	9	5
Median age (range)	51 (40–66)	58 (43–78)	59 (41–72)	46 (43–55)	69 (51–85)	61 (40–82)
Stages of cancer						
I	-	2	-	-	10	-
II	-	8	-	-	4	-
III	-	-	4	-	-	7
IV	-	-	6	-	-	9

### Immunoaffinity chromatography using MARS-14

An equal volume of individual plasma samples from each group (i.e. 10 non-metastatic CRC patients, NM; 10 metastatic CRC patients, M; and 10 healthy controls, HC) in the study-cohort were pooled. The pooled plasma samples were fractionated by MARS™ Multiple Affinity Removal Column, 4.6 x 100 mm, Hu-14 (MARS-14; Agilent Technologies, USA) using Agilent 1260 Infinity high performance liquid chromatography (HPLC; Agilent Technologies, USA) system to separate the 14 most abundant proteins (albumin, immunoglobulin G, immunoglobulin A, immunoglobulin M, transferrin, haptoglobin, antitrypsin, fibrinogen, alpha-2-macroglobin, alpha-1-acid glycoprotein, apolipoprotein A1, apolipoprotein A2, complement C3, and transthyretin) from the low-abundant proteins according to the manufacturer’s instructions. Briefly, 40 µl of pooled plasma were mixed with MARS™ buffer A at 1:3 (v/v), filtered through a 0.22 µm Spin-X cartridge tube filter (Corning Life Sciences, USA), and clarified by centrifugation at 16,000 × g for 1 min at 4°C before use. The HPLC system was set up according to the manufacturer’s recommendations. In brief, the MARS-14 column was equilibrated with 100% buffer A at a flow rate of 0.125 mL/min for 10 min. A total of 75 µl diluted plasma was injected into the column and run with 100% buffer A at the same flow rate for 18 min, then the flow rate was changed to 1 mL/min for 2 min. The system was changed to 100% MARS™ buffer B (elution buffer) for 7 min and back to 100% of buffer A for column equilibration at the flow rate of 0.125 mL/min for 11 min. The protein signals were monitored with an absorbance of 280 nm. The whole protein peaks were collected as the flow-through (FT) and elute (EL) fractions for the differential expression analysis of low and high-abundant proteins, respectively. Both fractions were buffer-exchanged with 50 mM ammonium bicarbonate using Spin-X UF concentrators (5 kDa cutoff; Corning Life Sciences, USA) by centrifugation at 15,000 × g for 30 min at 4°C. The protein concentration was determined using Bradford protein assay (Bio-Rad Laboratories, USA) and stored at − 80°C until use.

### Sample preparation for LC-MS/MS analysis

Five micrograms of FT and EL samples were reduced with 10 mM dithiothreitol at 95˚C for 5 min and alkylated with 20 mM iodoacetamide in the dark at RT for 30 min. The proteins were digested with trypsin (Promega, USA) at ratio of 1:50 (trypsin:protein) w/w at 37˚C, overnight. The digestion was stopped by adding formic acid to a final concentration of 1% v/v. The digested peptides were cleaned up using ZipTip C18 (Merck Millipore, USA), dried by SpeedVac (Labconco, USA) and kept at -20˚C until further processing by LC-MS/MS.

### LC-MS/MS analysis

The nanoflow liquid chromatography (Thermo Fisher Scientific, Waltham, MA, USA) coupled with amaZon speed ETD (Bruker Daltonics, Germany) with a CaptiveSpray ion source was used for peptide analysis. The tryptic-digested peptide samples were injected into an Acclaim PepMap RSLC C18 column (75 µm i.d. x 150 mm). The separation was performed at 300 nL/min using 70 min of 1–50% acetonitrile gradient containing 0.1% formic acid. The mass spectrometer was operated in the positive ion mode with a spray voltage of 1,500 V, dry temperature of 150˚C, without nebulizer gas and mass range between 400‑1,400 m/z. The parameter was optimized at 922 m/z with ion charge count target of 400,000. The raw LC‑MS/MS data were processed using Bruker Compass version 1.4 (Bruker Daltonics, Germany). Each sample was analyzed in triplicate, providing three technical replicates per sample.

### Label-free relative quantification and identification of proteins

Label-free relative quantification was performed using Progenesis QI version 3.1 (Nonlinear Dynamics, USA) as previously described [10] with certain modifications. In brief, three technical replicated MS spectral data of each sample group (non-metastatic CRC, metastatic CRC, and healthy control groups) were aligned and compared to provide quantitative measurement of matched peptides among all MS runs. Filtering parameters for peptide ions included MS peaks acquired in the range of 50-3000 m/z and retention time of 5–60 min, with charge states of 2+, 3+, and 4+. The obtained peaks were exported to perform MS/MS identification against the UniProt human proteome database (release 2019_03) using in-house MASCOT Server v.2.4.0 (Matrix Science, USA). The search parameters were set as follows: 1.2 Da and 0.6 Da for peptide mass tolerance and MS/MS ion mass tolerance respectively; #^13^C as 0; instrument type as ESI-TRAP; fixed modification of carbamidomethyl at cysteine residue; variable modifications of methionine oxidation and N-terminal carbamidomethylation; 1 missed cleavage allowance; enzyme as trypsin; the limit of peptide charges as 2+, 3+, and 4+; and including decoy database. Identity threshold of the search results was adjusted to yield 1% false discovery rate (FDR).

The identified peptide ions were imported back to Progenesis QI to synchronize with their related ion intensity data. Peptide identification was refined with the score cut-off corresponding to 1% FDR. The results were exported for further refining to exclude uncertain peptide identification with the followings criteria: deletion of non-unique sequences and conflicting sequences (sequences from an MS/MS identification that could be assigned to more than one sequence); and spectral count less than 2 MS runs across all MS runs. The proteins containing at least 2 peptides were accounted for relative quantification.

### Functional annotation analysis

The functional analysis of the proteins with greater than 1.5-fold differential expression level between the CRC and the healthy control groups were analyzed for their biological processes (GOTERM_BP_DIRECT) and molecular functions (GOTERM_MF_DIRECT) using the Database for Annotation, Visualization and Integrated Discovery (DAVID) 2021 [11, 12] with its comprehensive knowledgebase (v2022q1). The functional classes with *p*-value < 0.05 and false discovery rate (FDR) < 0.05 were considered to be significantly related to the altered proteins in the particular CRC stages.

### Immunoblot analysis

To verify the quantitative proteomics results, immunoblot analysis of certain proteins including alpha-1-acid glycoprotein 1 (AGP1), alpha-1-antitrypsin (A1AT), complement component C9 (C9), fibronectin (FN), leucine-rich alpha-2-glycoprotein-1 (LRG) and protein S100-A8 (S100A8) was performed. Three sets of immunoblotting were performed, consisting of [1] the pooled samples in the study-cohort used for label-free quantitative proteomics (n = 3), individual samples in [2] the study-cohort (n = 30), and [3] the validation-cohort (n = 45). Equal amount of protein samples was separated by 10% TGX Stain-Free FastCast (Bio-Rad, USA). The total protein levels of each sample were visualized and calculated using stain-free imaging in the Gel Doc™ EZ Imager (Bio-Rad, USA) and then transferred onto PVDF membranes (Merck Millipore, USA). The membranes were blocked in 3% bovine serum albumin (BSA) in TBS/T for 1 hour and probed with primary monoclonal antibodies (Abcam, UK); anti-AGP1 (ab134160; 1:1,000), anti-A1AT (ab167414; 1:1,000), anti-C9 (ab173302; 1:2,000), anti-FN (ab32419; 1:2,000), anti-LRG (ab178698; 1:1,000), and anti-S100A8 (ab92331; 1:1,000) at 4˚C overnight. The membranes were then incubated with anti-rabbit secondary antibody conjugated with horseradish peroxidase (HRP) (P0217, Dako, Denmark; 1:5,000) in 5% skim milk in TBS/T at RT for 1 hour. The membranes were washed 15 minutes, 3 times, with TBS/T after primary and secondary antibody incubations. The immunoblots were reacted with SuperSignal™ Western Blot Substrate (Thermo Scientific, USA). The band intensities were visualized and quantified by the ImageQuant LAS 4000 digital imaging system (GE Healthcare, USA). The expression level of a particular protein in the sample was normalized by the total protein intensity obtained from stain-free imaging before use in relative quantification and statistical analysis

### Evaluation of the diagnostic performance of biomarker candidates

The expression levels of proteins obtained from protein band intensities of immunoblotting were used for receiver operating characteristic (ROC) curve analysis and performed using Prism version 9 (GraphPad Software, USA). For each biomarker candidate, the normalized expression level was directly analyzed using ROC curve function. However, for the combination of biomarkers candidates, ROC curve was generated using the predicted probability derived from the binary logistic regression of the normalized expression level of a set of 3 biomarker candidates. Sensitivity and specificity represented the diagnostic performance of each combination and the set of biomarker candidates were selected from where the cut-off gave the highest sum of sensitivity and specificity.

### Statistical analysis

Statistical analyses were performed using Prism version 9 (GraphPad Software, USA). The differences among the healthy control, non-metastatic CRC and metastatic CRC groups were calculated using Kruskal-Wallis (non-parametric one-way ANOVA) and Dunn’s post hoc tests, while the differences between the healthy control group and the CRC (CRC patients including non-metastatic-staged and metastatic-staged patients) group were calculated by Mann-Whitney U test (non-parametric *t*-test) or Welch’s *t*-test (parametric *t*-test), as appropriate. Differences were considered statistically significant at *p*-value < 0.05.

## Results

### Identification of high- and low-abundant plasma proteins using label-free quantitative proteomics

**Fig. 1:**
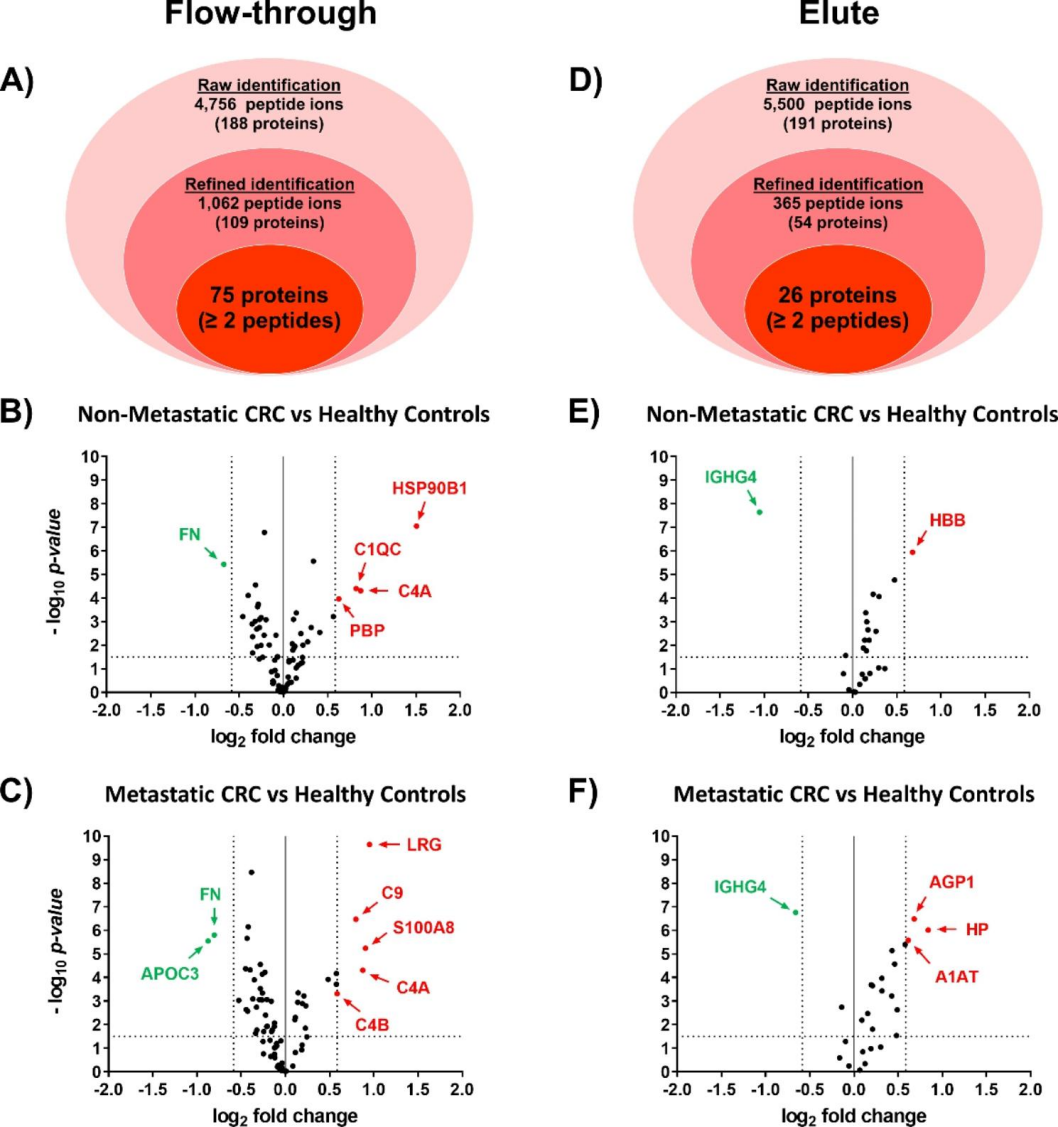
Infographic of plasma proteins identified by label-free quantitative proteomics in flow-through (**A**) and elute (**D**) fractions of pooled plasma from healthy controls, non-metastatic CRC patients and metastatic CRC patients separated by MARS-14 immunochromatography. Volcano plots of the protein expression levels in non-metastatic and metastatic CRC patients in comparison to those of the healthy controls in the flow-through (**B** and **C**) and elute (**E** and **F**) fractions, respectively. The x-axis represents log_2_ fold changes of proteins and the y-axis represents -log_10_*p*-value. The red and green dots indicate proteins with significantly different expression identified by log_2_ fold change > 0.58 and < -0.58 (> 1.5-fold differential expression), and -log_10_*p*-value greater than 1.3 (*p*-value < 0.05), respectively. The black dots indicate proteins which were not significantly altered between CRC patients and healthy control groups.

The low-abundant proteins and the 14 most-abundant proteins in blood plasma samples of each group (pooled samples) in the study-cohort were efficiently separated into FT and EL fractions, respectively, using MARS-14 immunoaffinity chromatography. Label-free quantitative proteomics analysis of these fractions revealed identification of 4,756 and 5,500 peptide ions which corresponded to 188 and 191 proteins in FT and EL fractions, respectively. After refining with score cut-off and deletion of uncertain identification across MS runs, there were 1062 and 365 peptide ions in FT and EL fractions, respectively. A total of 75 proteins in FT fraction and 26 proteins in EL fraction have at least 2 identified peptide sequences per protein, and thus were good for label-free quantitative analysis (Fig. 1A and 1D). The full detail of quantitative proteomics analyses is shown in the supplementary data, Table S2. Differential expression analysis of the FT fraction between CRC and healthy control groups showed that 4 proteins (HSP90B1, C4A, C1qC, and PBP) were up-regulated and 1 protein (FN) was down-regulated in non-metastatic CRC, while 5 proteins (LRG, S100A8, C4A, C9, and C4B) were up-regulated and 2 proteins (APOC3 and FN) were down-regulated in metastatic CRC groups, respectively (Fig. 1B and 1C). In the EL fraction, 1 protein (HBB) was up-regulated and 1 protein (IGHG4) was down-regulated in non-metastatic CRC, while 3 proteins (HP, AGP1, and A1AT) were up-regulated and 1 protein (IGHG4) was down-regulated in the metastatic CRC group, respectively (Fig. 1E and 1F). The list of proteins showing log_2_ fold change greater than +/-0.58 (> 1.5 fold differentially expressed level) in patients with either non-metastatic or metastatic CRC compared to those of the healthy controls were shown in Table 2.

**Table 2:** The list of proteins exhibited by log_2_ fold change > 0.58 and < -0.58 (> 1.5-fold differential expression) in either non-metastatic or metastatic CRC patients compared to healthy controls.

	UniProt accession no.	Gene Name	Protein Name	Identified peptides	Fold change (log_2_)^#^		
					NM vs HC	M vs HC	
*The flow-through fraction*							
	P02750	LRG1	Leucine-rich α-2-glycoprotein (LRG)	7	0.34^***^ (0.31 to 0.36)	0.95^****^ (0.95 to 0.95)	
	P05109	S100A8	Protein S100-A8 (S10A8)	2	-0.12 (-0.17 to -0.08)	0.91^***^ (0.84 to 0.98)	
	P0C0L4	C4A	Complement C4-A (CO4A)	2	0.87^***^ (0.82 to 0.92)	0.88^***^ (0.77 to 0.94)	
	P02748	C9	Complement component C9 (CO9)	14	0.20^**^ (0.17 to 0.23)	0.80^***^ (0.77 to 0.84)	
	P0C0L5	C4B	Complement C4-B (CO4B)	2	0.56^**^ (0.50 to 0.62)	0.59^***^ (0.52 to 0.69)	
	P02775	PPBP	Platelet basic protein (CXCL7)	4	0.63^***^ (0.58 to 0.66)	0.58^**^ (0.53 to 0.65)	
	P14625	HSP90B1	Endoplasmin (ENPL)	2	1.51^****^ (1.49 to 1.53)	-0.08 (-0.17 to 0.07)	
	P02747	C1QC	Complement C1q subcomponent subunit C (C1QC)	3	0.82^**^ (0.77 to 0.85)	-0.09 (-0.18 to -0.04)	
	P02751	FN1	Fibronectin (FN)	25	-0.67^**^ (-0.69 to -0.64)	-0.80^**^ (-0.82 to -0.79)	
	P02656	APOC3	Apolipoprotein C-III (APOC3)	2	-0.16^*^ (-0.18 to -0.15)	-0.87^***^ (-0.91 to -0.81)	
***The elute fraction***							
	P00738	HP	Haptoglobin (HP)	9	0.26^*^ (0.24 to 0.29)	0.84^****^ (0.80 to 0.88)	
	P02763	ORM1	alpha-1-acid glycoprotein 1 (AGP1)	4	0.30^**^ (0.26 to 0.36)	0.68^****^ (0.67 to 0.69)	
	P01009	SERPINA1	alpha-1-antitrypsin (A1AT)	11	0.47^**^ (0.41 to 0.52)	0.61^***^ (0.60 to 0.64)	
	P68871	HBB	Hemoglobin subunit β (HBB)	6	0.68^***^ (0.65 to 0.70)	0.58^**^ (0.56 to 0.59)	
	P01861	IGHG4	Immunoglobulin heavy constant γ4 (IGHG4)	3	-1.05^****^ (-1.10 to -0.99)	-0.66^****^ (-0.68 to -0.64)	

^#^ Fold changes were calculated from the average normalized ratio of total MS intensity of all peptides obtained from technical triplicate MS/MS runs from pooled samples (n = 10) of each CRC and HC groups. Values in parentheses represent the range of fold changes calculated from technical triplicates. NM, non-metastatic CRC patients; M, metastatic CRC patients, HC, healthy controls. *, **, ***, and **** represent *p*-value < 0.05, < 0.01, < 0.001, and < 0.0001, respectively. The full data of statistical analysis was shown in the supplementary Table S2.

## Functional analysis of the differentially expressed plasma proteins in CRC patients

According to the complete gene ontology (GO) database provided by http://geneontology.org/ (accessed on April 28, 2022), all proteins exhibited greater than 1.5-fold differential expression level (log_2_ fold change greater than +/-0.58) were involved in various GO classes (the full detail of analyses was in the supplementary data, Table S3). To highlight the GO classes which may play important roles in the different CRC conditions, the functional annotation analysis of the differentially expressed proteins was carried out by DAVID 2021 functional annotation tool. The results were shown in Table 3 and the supplementary data, Table S3. The proteins in which their expressions were highly altered in the non-metastatic CRC group were involved in complement activation, classical pathway (GO:0006958) whereas those in the metastatic CRC group were clustered in acute-phase response (GO:0006953), complement activation, classical pathway (GO:0006958), and inflammatory response (GO:0006954). The more GO classes found may represent the more pathobiological changes related to CRC cancer progression.

**Table 3:** Functional annotation clustering of the proteins showing greater than 1.5-fold differential expression level between CRC patients and healthy controls. The gene ontology (GO) classes with *p*-value < 0.05 and false discovery rate (FDR) < 0.05 were considered significant.

GO Accession	Term | Involved proteins	*p*-value	FDR
*Non-Metastatic CRC Group*			
GO:0006958	Complement activation, classical pathway | C1qC, IGHG4 and C4A	< 0.001	0.0427
GO:0045087	Innate immune response | C1qC, IGHG4 and C4A	0.013	0.3363
GO:0070527	Platelet aggregation | HBB and FN	0.014	0.3363
GO:0042742	Defense response to bacterium | IGHG4, PBP	0.070	1.000
***Metastatic CRC group***			
GO:0006953	Acute-phase response | A1AT and AGP1, FN and HP	< 0.001	0.0001
GO:0006958	Complement activation, classical pathway | C4B, IGHG4, C4A and C9	< 0.001	0.0017
GO:0006954	Inflammatory response | C4B, C4A, AGP1 and S100A8	< 0.001	0.0377
GO:0010951	Negative regulation of endopeptidase activity | A1AT, C4B and C4A	0.003	0.0594
GO:0045087	Innate immune response | C4B, IGHG4, C4A and S100A8	0.003	0.0594
GO:2000427	Positive regulation of apoptotic cell clearance | C4B and C4A	0.003	0.0594
GO:0042742	Defense response to bacterium | IGHG4, HP and S100A8	0.006	0.0979
GO:0002526	Acute inflammatory response | HP and S100A8	0.009	0.1251
GO:0006956	Complement activation | C4B and C4A	0.010	0.1251
GO:0004866	Endopeptidase inhibitor activity | C4B and C4A	0.023	0.8819
GO:0002020	Protease binding | A1AT and FN	0.056	1.0000

### Verification of proteins identified by label-free quantitative proteomics using immunoblotting

**Fig. 2:**
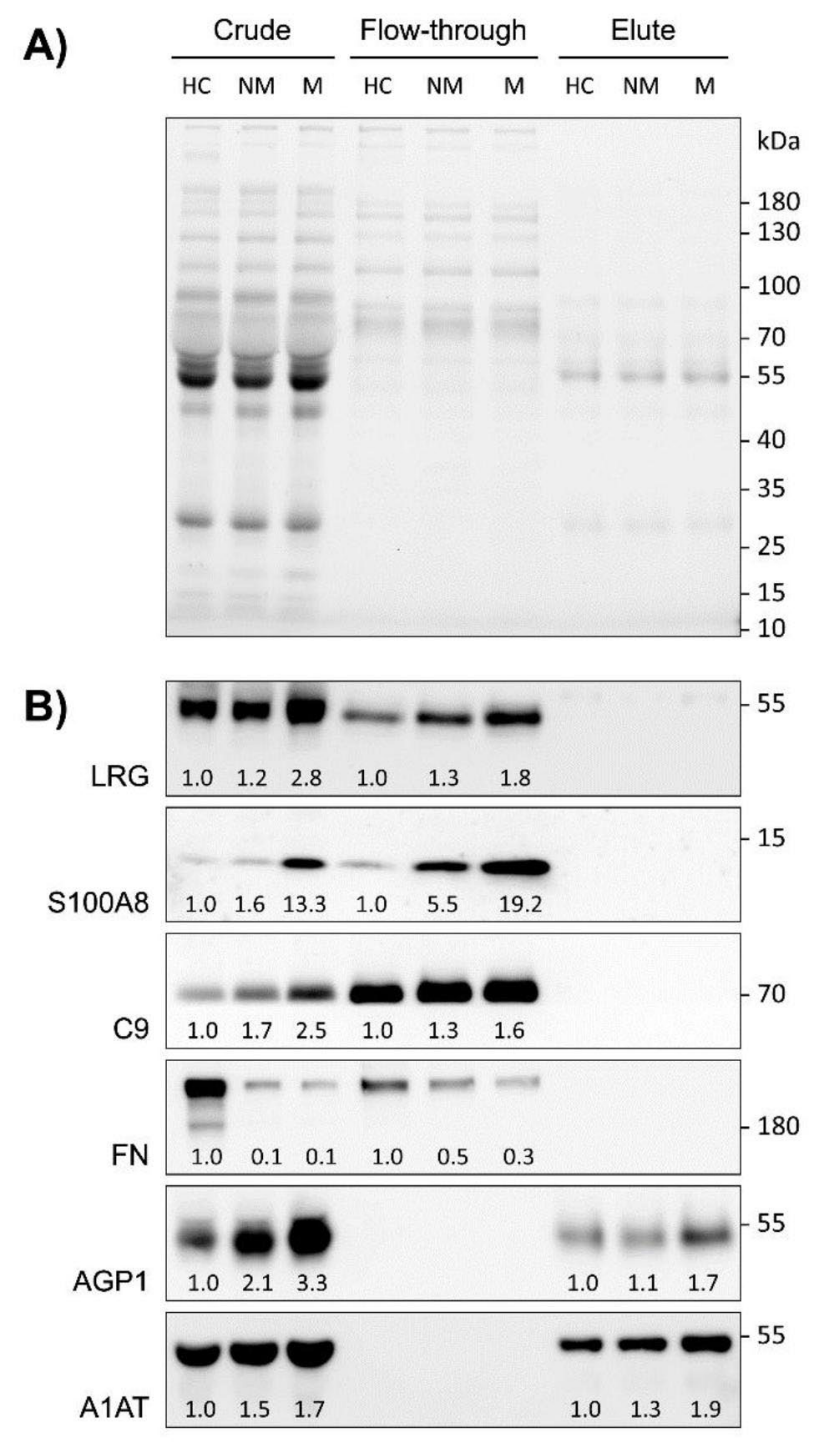
The protein patterns and expression levels of LRG, S100A8, C9, FN, AGP1, and A1AT in crude plasma, MARS-14 flow-though (FT) and MARS-14 elute (EL) fractions of the pooled samples of healthy controls (HC), patients with non-metastatic CRC (NM) and patients with metastatic CRC (M). (**A**) Stain-free imaging of the gel displayed the pattern of total proteins from three groups (HC, NM, and M). Crude plasma (30 µg), flow-through (5 µg) and elute (5 µg) fractions of three groups were separated on 10% TGX stain-free FastCast. Total proteins were visualized by a stain-free imaging system. (**B**) Immunoblots of LRG, S100A8, C9, FN, AGP1, and A1AT. Values below immunoblots denote the ratio of each protein band intensity normalized by its total protein loading and compared to those of the healthy control.

The pooled samples in the study-cohort used for label-free quantitative proteomics, including crude plasma, FT, and EL fractions of healthy controls (HC), of patients with non-metastatic CRC (NM) and of patients with metastatic CRC (M) were also subjected to immunoblotting to verify some of the markers with high changes in their expression levels. Except for haptoglobin which was excluded from the study due to unavailability of a proper primary antibody to discriminate between haptoglobin and haptoglobin-related protein, the expression levels of the 2 most differentially expressed proteins from each fraction based on metastatic group, i.e. LRG, S100A8, AGP1, and A1AT (Table 2), as well as C9 and FN which showed results consistent with our previous glycoproteomic studies [10], were confirmed in the pooled samples of crude plasma, FT and EL fractions by immunoblotting. The protein patterns under stain-free imaging of the gel revealed the removal of high-abundant proteins that were absent in the FT fraction but present in the EL fraction in comparison to crude plasma (Fig. 2A). With a similar amount protein loading as seen by the stain-free imaging of the gel, the intensity of immunoblot signals of these proteins was normalized by its total protein-loading intensity and further compared to those of the healthy control of each sample type (crude plasma, FT, and EL fractions). In the FT fraction, the levels of LRG, S100A8, C9 were increased while FN was decreased in CRC stage-dependent manner (non-metastatic and metastatic stages), when compared with those of the healthy control (Fig. 2B). In the EL fraction, the levels of AGP1 and A1AT were increased in CRC stage-dependent manner (Fig. 2B). The expression level of these proteins as determined by the label-free quantitative proteomics and immunoblotting were in a similar manner except for the level of S100A8 found in the FT fraction of NM-CRC group, which was slightly changed in MS analysis but greatly increased in immunoblot detection. Besides the FT and EL fractions, the levels of these proteins were also detected in the crude plasma samples. The results showed increased levels of LRG, S100A8, C9, AGP1 and A1AT, and decreased level of FN in a CRC stage-dependent manner, similar to that found in fractionated samples. These results confirmed the use of crude plasma for preliminarily screening of individual samples to determine whether these proteins could be protein candidates for CRC detection.

### Validation of biomarker candidates in CRC patients

**Fig. 3:**
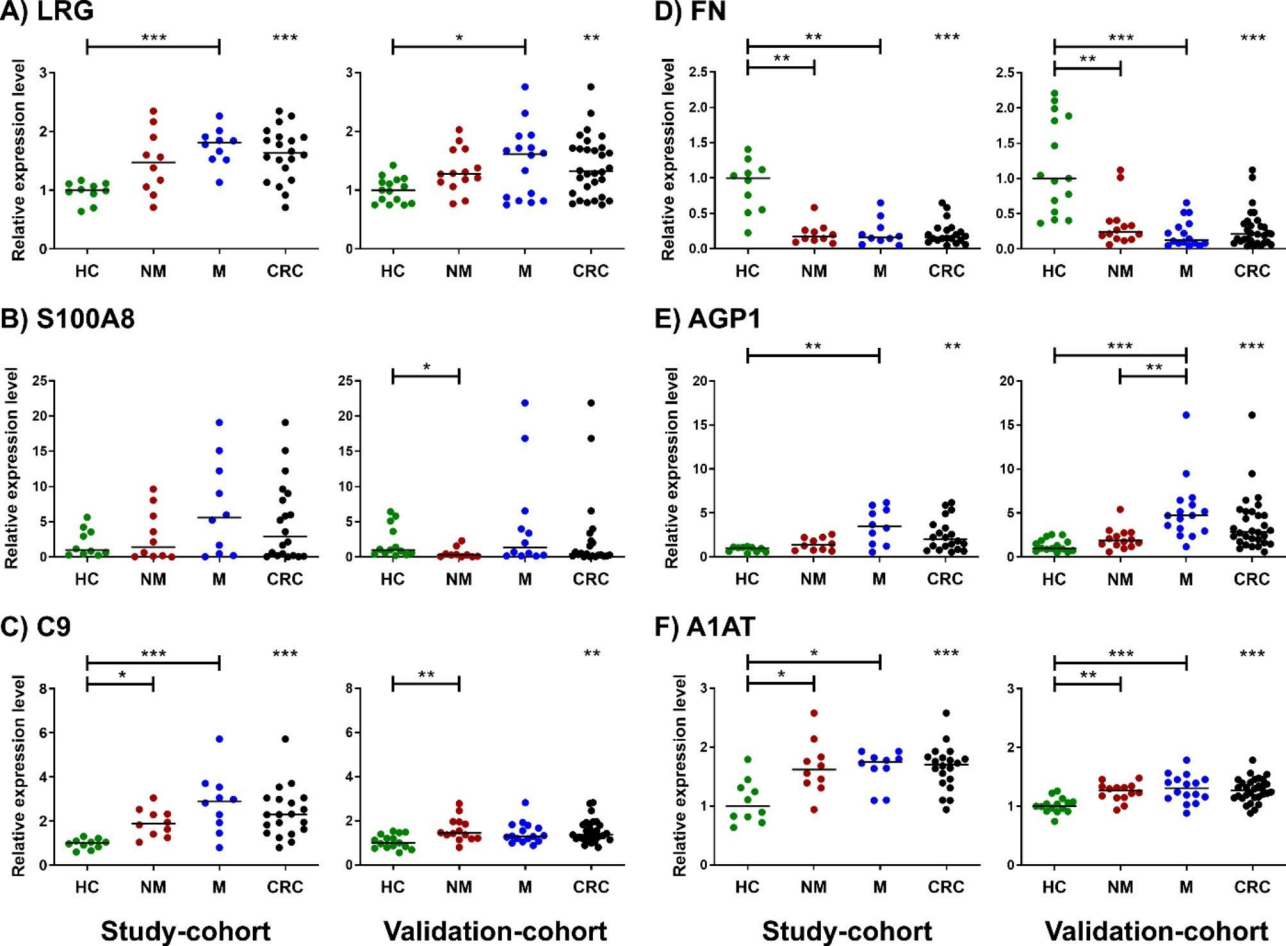
Scatter plots show relative expression levels of (**A**) LRG, (**B**) S100A8, (**C**) C9, (**D**) FN, (**E**) AGP1, and (**F**) A1AT from immunoblotting of individual plasma samples in the study-cohort and validation-cohort. All immunoblot results are provided in the supplementary data, Table S4 and S5. Green dots, Healthy control (HC); Red dots, Non-Metastatic CRC patients (NM); Blue dots, Metastatic CRC patients (M); Black dots, all CRC patients (CRC). Black lines represent the medians of samples in each group. Stars represent statistical significance calculated by non-parametric one-way ANOVA (Kruskal-Wallis) and Dunn’s multiple comparison test for the comparison among HC, NM and M groups; and non-parametric *t*-test (Mann-Whitney U test) for the comparison between HC and CRC groups. *, **, and *** represent *p-*value < 0.05, < 0.01, and < 0.001, respectively.

The expression levels of LRG, S100A8, C9, FN, AGP1, and A1AT were further validated in individual plasma samples of 2 independent cohorts by immunoblotting. The first cohort (study-cohort) contained plasma samples from individuals whose plasma was used for the pooled samples in the early experiments, while the second cohort (validation-cohort) contained another set of plasma samples unrelated to the first cohort. The quantitative results were shown in Fig. 3 (the full detail of analyses was in the supplementary data, Table S4 for the study-cohort and S5 for the validation-cohort).

LRG (Fig. 3A) – In the study-cohort, the relative expression levels were increased in both non-metastatic and metastatic patients (median = 1.471 and 1.807 folds; *p*-value > 0.05 and < 0.001, respectively) compared to healthy controls. In the validation-cohort, the relative expression level was also increased in both non-metastatic and metastatic patients (median = 1.280 and 1.641 folds; *p*-value > 0.05 and < 0.05, respectively) compared to healthy controls. When combining all CRC patients together, the expression levels were increased in both cohorts with statistically significant difference of *p*-value < 0.001 and < 0.01, respectively. Of note, patients with CRC metastasis in the validation-cohort showed a bimodal pattern; some had low-expression while some had high-expression level of LRG. Based on their clinical data, there was no correlation of the LRG levels versus their sex, age and stage. Other clinical data should be investigated to see if any other factor correlates with the LRG level.

S100A8 (Fig. 3B) - In the study-cohort, the relative expression level was increased in both non-metastatic and metastatic patients (median = 1.402 and 5.603 folds, respectively) compared to healthy controls, but no statistical significance was presented in any aspect. In the validation-cohort, the results showed discrepancy with the study-cohort. It was increasing in non-metastatic patients but slightly increasing in metastatic patients (median = 0.286 and 1.358 folds; *p*-value < 0.05 and > 0.05, respectively). Noteworthy, in this study, S100A8 level had a very wide range of expression. Some patients had an extremely high level of S100A8 expression while some were undetectable. Although the longer exposure time on immunoblot was performed, none of signal was obtained. This is likely to be a limitation of immunoblotting for proteins presented with very low amount. Therefore, S100A8 was unlikely to be a reliable candidate for CRC biomarker.

C9 (Fig. 3C) – In the study-cohort, the relative expression levels were increased in both non-metastatic and metastatic patients (median = 1.884 and 2.898 folds; *p*-value < 0.05 and < 0.001, respectively) compared to healthy controls. In the validation-cohort, the relative expression level was also increased in both non-metastatic and metastatic patients (median = 1.447 and 1.300 folds; *p*-value < 0.01 and > 0.05, respectively) compared to healthy controls. When combining all CRC patients together, the expression levels were increased in both cohorts with statistically significant difference of *p*-value < 0.001 and < 0.01, respectively.

FN (Fig. 3D) – In the study-cohort, the relative expression levels were decreased in both non-metastatic and metastatic patients (median = 0.172 and 0.164 folds; *p*-value < 0.01 and < 0.01, respectively) compared to the healthy controls. In the validation-cohort, the relative expression level was also decreased in both non-metastatic and metastatic patients (median = 0.241 and 0.128 folds; *p*-value < 0.01 and < 0.001, respectively) compared to the healthy controls. When combining all CRC patients together, the expression levels were decreased in cohorts with statistically significant difference of *p*-value < 0.001 and < 0.001, respectively.

AGP1 (Fig. 3E) – In the study-cohort, the relative expression levels were increased in both non-metastatic and metastatic patients (median = 1.379 and 3.478 folds; *p*-value > 0.05 and < 0.01, respectively) compared to the healthy controls. In the validation-cohort, the relative expression level was also increased in both non-metastatic and metastatic patients (median = 1.878 and 4.718 folds; *p*-value > 0.05 and < 0.001, respectively) compared to the healthy controls. When combining all CRC patients together, the expression levels were increased in both cohorts with statistically significant difference of *p*-value < 0.01 and < 0.001, respectively. Interestingly, AGP1 level was distinguished between two disease stages with a statistically significant difference in the validation-cohort and likely to be increased in the metastatic group of the study-cohort.

A1AT (Fig. 3F) – In the study-cohort, the relative expression levels were increased in both non-metastatic and metastatic patients (median = 1.663 and 1.644 folds; *p*-value < 0.05 and < 0.05, respectively) compared to healthy controls. In the validation-cohort, the relative expression level was also increased in both non-metastatic and metastatic patients (median = 1.265 and 1.309 folds; *p*-value < 0.01 and < 0.001, respectively) compared to the healthy controls. When combining all CRC patients together, the expression levels were increased in both cohorts with statistically significant difference of *p*-value < 0.001 and < 0.001, respectively.

### ROC analysis of biomarker candidates to discriminate between CRC patients and healthy controls

**Fig. 4:**
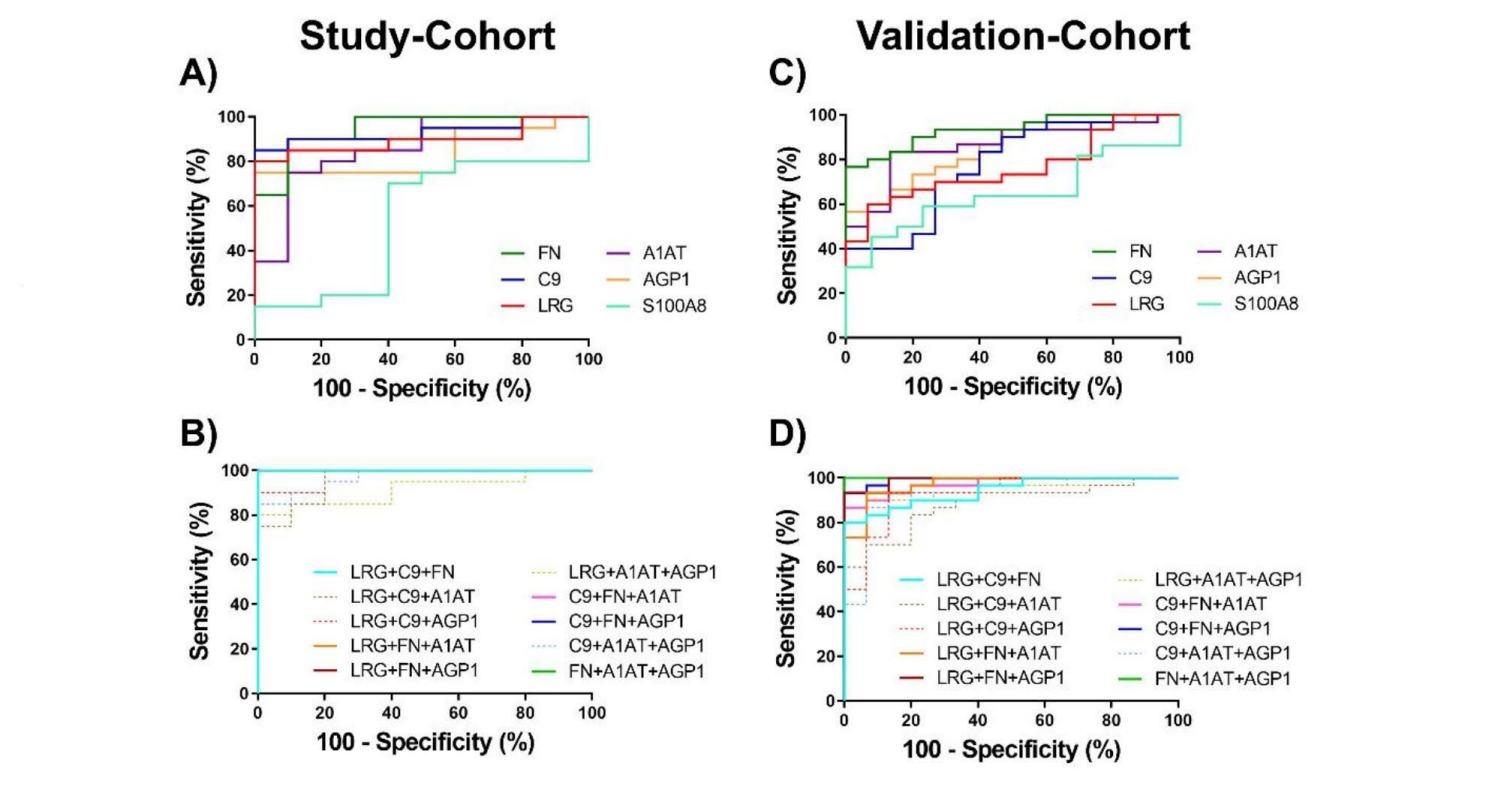
ROC curves representing the diagnostic performance of biomarker candidates between CRC patients and healthy controls. The upper charts demonstrated ROC curves of each biomarker candidate in (**A**) the study-cohort and (**C**) the validation-cohort. The bottom charts demonstrated ROC curves of the combination sets in (**B**) the study-cohort and (**D**) the validation-cohort. Details of the area under the ROC curve (AUC) with 95% confidence interval (CI), sensitivity and specificity of each protein candidate and the most effective combinations were shown in the Table 4.

To investigate the diagnostic performances of biomarker candidates for CRC detection, ROC curve analyses were performed. Figure 4 and Table 4 showed the diagnostic performances of biomarker candidates as a single and combination use in the study-cohort and validation-cohort.

In the study-cohort (Fig. 4A and 4B, Table 4), FN exhibited the best diagnostic performance with AUC of 0.945, 90% sensitivity and 90% specificity; followed by C9 (AUC = 0.930, 85% sensitivity and 100% specificity), LRG (AUC = 0.895, 80% sensitivity and 100% specificity), A1AT (AUC = 0.860, 75% sensitivity and 90% specificity), AGP1 (AUC = 0.835, 75% sensitivity and 100% specificity), and S100A8 (AUC = 0.535, 70% sensitivity and 60% specificity), respectively. Due to the poor discriminatory power of S100A8 shown in the study-cohort, S100A8 was excluded from the combined biomarker panels in further evaluation. Interestingly, the combination of 3 biomarker candidates demonstrated greatly improved diagnostic performance compared to individual tests. The following combinations conferred perfect discriminatory power between the healthy and CRC patient groups (AUC = 1.000, 100% sensitivity and 100% specificity), including LRG + C9 + FN, LRG + FN + A1AT, LRG + FN + AGP1, C9 + FN + A1AT, C9 + FN + AGP1, and FN + A1AT + AGP1. Other combinations also exhibited improved performances compared to the use of single candidate (Table 4). The full detail of diagnostic performance was shown in the supplementary data, Table S6.

In the validation-cohort (Fig. 4C and 4D, Table 4), FN still exhibited the best diagnostic performance with AUC of 0.933, 77% sensitivity and 100% specificity. The next most effective biomarker candidates in the validation-cohort were A1AT with AUC of 0.862, 83% sensitivity and 87% specificity, followed by AGP1 (AUC = 0.849, 57% sensitivity and 100% specificity), C9 (AUC = 0.778, 70% sensitivity and 73% specificity) and LRG (AUC = 0.762, 60% sensitivity and 93% specificity), respectively. Again, S100A8 exhibited a poor diagnostic performance with AUC of 0.647, 45% sensitivity and 92% specificity. For the diagnostic performance of combined biomarker candidates, FN + A1AT + AGP1 exhibited the best diagnostic performance with perfect discriminatory power (AUC = 1.000, 100% sensitivity and 100% specificity). Other combination models demonstrated very good diagnostic performance with AUCs above 0.9 except for LRG + C9 + A1AT which had an AUC of 0.880. The full detail of diagnostic performance was shown in the supplementary data, Table S6.

**Table 4:** Diagnostic performance of individual biomarker candidates and their combination in distinguishing CRC patients from healthy controls in the study- and validation-cohorts.

Biomarker	Study-Cohort					Validation-Cohort		
	AUC (95% CI)	Sensitivity (%)	Specificity (%)		AUC (95% CI)		Sensitivity (%)	Specificity (%)
FN***	0.945 (0.864-1.000)	90	90		0.933 (0.865-1.000)		77	100
C9**	0.930 (0.835-1.000)	85	100		0.778 (0.634–0.922)		70	73
LRG**	0.895 (0.778-1.000)	80	100		0.762 (0.624-0.900)		60	93
A1AT**	0.860 (0.716-1.000)	75	90		0.862 (0.753–0.972)		83	87
AGP1**	0.835 (0.689–0.981)	75	100		0.849 (0.738–0.960)		57	100
**S100A8**	0.535 (0.304–0.766)	70	60		0.647 (0.465–0.828)		45	92
FN + A1AT + AGP1***	1.000 (1.000–1.000)	100	100		1.000 (1.000–1.000)		100	100
C9 + FN1 + AGP1***	1.000 (1.000–1.000)	100	100		0.993 (0.979-1.000)		93	100
LRG + FN + AGP1***	1.000 (1.000–1.000)	100	100		0.991 (0.973-1.000)		93	100
C9 + FN + A1AT***	1.000 (1.000–1.000)	100	100		0.973 (0.936-1.000)		87	100
LRG + FN + A1AT***	1.000 (1.000–1.000)	100	100		0.971 (0.928-1.000)		93	93
LRG + C9 + FN***	1.000 (1.000–1.000)	100	100		0.942 (0.880-1.000)		80	100
LRG + C9 + AGP1***	0.980 (0.940-1.000)	90	100		0.929 (0.849-1.000)		93	87
C9 + A1AT + AGP1***	0.970 (0.919-1.000)	90	90		0.931 (0.847-1.000)		87	93
LRG + C9 + A1AT***	0.960 (0.895-1.000)	100	80		0.880 (0.718–0.979)		70	93
LRG + A1AT + AGP1***	0.915 (0.814-1.000)	80	100		0.922 (0.836-1.000)		90	93

Note: The sensitivity and specificity were derived from the point on the ROC curve where it gave the highest sum of sensitivity and specificity. *, **, and *** represent *p-*value < 0.05, < 0.01, and < 0.001, respectively.

### ROC analysis of biomarker candidates to discriminate between the patients with NM-CRC and M-CRC stages

**Fig. 5:**
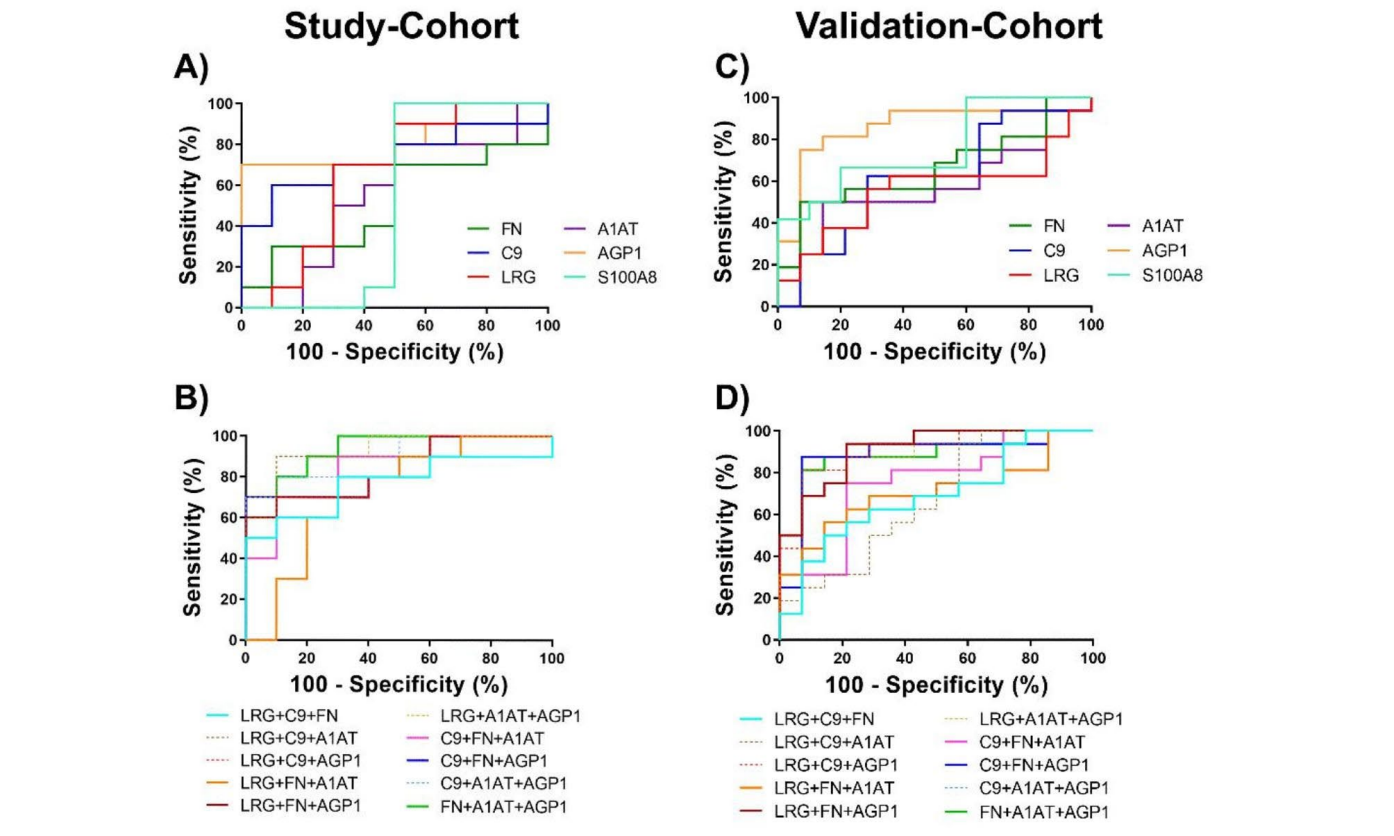
ROC curves representing the diagnostic performance of biomarker candidates between CRC patients with non-metastatic and metastatic stages. The upper charts demonstrated ROC curves of each biomarker candidate in (A) the study-cohort and (C) the validation-cohort. The bottom charts demonstrated ROC curves of the combination sets in (B) the study-cohort and (D) the validation-cohort. Details of the area under the ROC curve (AUC) with 95% confidence interval (CI), sensitivity and specificity of each protein candidate and the most effective combinations were shown in the Table 5.

When CRC subgroups further analyzed (Fig. 5 and Table 5), all individual biomarkers demonstrated poor diagnostic performance in distinguishing metastasis CRC (M-CRC) from non-metastatic (NM-CRC) patients except for AGP1. It showed a good diagnostic performance for differentiation between M-CRC and NM-CRC cases in both the study-cohort (AUC = 0.790, 70% sensitivity and 100% specificity) and the validation-cohort (AUC = 0.866, 75% sensitivity and 93% specificity). Furthermore, compared with their individual use, the combined use of three-biomarkers improved AUCs and were able to differentiate M-CRC from NM-CRC. In the study-cohort; FN + A1AT + AGP1, LRG + FN + AGP1, C9 + FN + A1AT, and C9 + FN + AGP1 performed a good capability in discriminating M-CRC from NM-CRC with AUCs > 0.8. While in the validation-cohort, only LRG + FN + AGP1, C9 + FN + AGP1 and FN + A1AT + AGP1 exhibited a good diagnostic performance with AUCs at least 0.8 to discriminate M-CRC from NM-CRC. The full detail of diagnostic performance was shown in the supplementary data, Table S7.

**Table 5:** Diagnostic performance of individual biomarker candidates and their combination in distinguishing between NM-CRC and M-CRC patients in the study- and validation-cohorts.

Biomarker	Study-Cohort			Validation-Cohort		
	AUC (95% CI)	Sensitivity (%)	Specificity (%)	AUC (95% CI)	Sensitivity (%)	Specificity (%)
**FN**	0.510 (0.245–0.775)	30	90	0.661 (0.462–0.860)	20	93
**C9**	0.730 (0.496–0.964)	60	90	0.616 (0.408–0.824)	63	71
**LRG**	0.660 (0.405–0.915)	70	70	0.558 (0.343–0.773)	56	71
**A1AT**	0.550 (0.283–0.817)	80	50	0.580 (0.370–0.791)	44	86
AGP1*	0.790 (0.567-1.00)	70	100	0.866 (0.727-1.000)	75	93
**S100A8**	0.510 (0.205–0.815)	100	50	0.758 (0.554–0.962)	67	80
FN + A1AT + AGP1**	0.920 (0.800-1.000)	80	90	0.875 (0.740-1.000)	81	93
C9 + FN1 + AGP1*	0.810 (0.598-1.000)	70	90	0.884 (0.746-1.000)	88	93
LRG + FN + AGP1*	0.840 (0.664-1.000)	70	90	0.911 (0.809-1.000)	94	78
C9 + FN + A1AT*	0.810 (0.601-1.000)	70	90	0.755 (0.577–0.933)	75	79
**LRG + FN + A1AT**	0.730 (0.496–0.964)	80	70	0.705 (0.515–0.896)	44	93
**LRG + C9 + FN**	0.770 (0.549–0.992)	60	90	0.688 (0.495–0.880)	50	86
**LRG + C9 + A1AT**	0.870 (0.678-1.000)	60	90	0.674 (0.477–0.872)	94	43
LRG + C9 + AGP1*	0.820 (0.633-1.000)	70	90	0.906 (0.798-1.000)	81	93
LRG + A1AT + AGP1***	0.940 (0.840-1.000)	90	90	0.897 (0.784-1.000)	81	93
C9 + A1AT + AGP1**	0.910 (0.783-1.000)	80	90	0.880 (0.744-1.000)	81	93

Note: The sensitivity and specificity were derived from the point on the ROC curve where it gave the highest sum of sensitivity and specificity. *, **, and *** represent *p-*value < 0.05, < 0.01, and < 0.001, respectively.

## Discussion

### Identification of plasma glycoproteins in CRC patients

Human biological fluids including blood plasma samples contain various proteins and some of which may reflect the prediction of physio-pathological conditions and disease. MS-based proteomics has been widely used as a strategy not only for the identification of protein biomarkers but also for the quantification of targeted proteins in biological fluids for clinical application [13]. However, biomarker discovery using plasma samples is challenging because plasma contains various proteins of different concentration. A number of research attempts to identify blood-based protein biomarkers for CRC and several potential proteins have been proposed. For example, Gao et al., reported the evaluation of CEA, CA19-9, CA72-4, CA125, and ferritin in serum samples of CRC and found that the combination of these biomarkers were positively correlated with invasion and tumor-node-metastasis stages [14]. Harlid et al. showed that two potential biomarkers, FGF-21 and PPY, were positively associated to colon and rectal cancer risk [15]. However, they suggested that single biomarkers may not be effective for CRC screening. Ahn et al., reported that the levels of seven proteins (CST3, GPX3, CFD, MRC1, COMP, PON1, and ADAMDEC1) were altered in plasma samples of patients with CRC stage I-IV in comparison to those of healthy controls using affinity chromatography (e.g. MARS-14) and Sequential Window Acquisition of All Theoretical Mass Spectra (SWATH-MS) [6]. However, using western blotting and/or enzyme-linked immunosorbent assay (ELISA) for validation, only some proteins reveled consistent with the SWATH-MS results. All these finding indicate that low- and high-abundant proteins presented in blood could possibly be targeted for seeking of potential CRC biomarkers. Herein, MARS14 affinity chromatography was used to fractionate low- and high-abundant proteins from the pooled plasma samples of patients with NM-CRC and M-CRC as well as those of healthy controls. Our proteomics strategy was performed, using [1] identification and quantification of potential marker candidates by detecting changes of proteins in the pooled samples between three groups and [2] verification of some protein markers showing large changes in their expression levels from all individual samples. Although some potential candidates may have been missed using sample pooling, it is assumed that by pooling, typical biological variations among individuals are generally averaged out, and so that the majority of candidates are of similar concentrations within each group. In addition, the label-free quantitative proteomics analysis of many individual samples was challenging, involving chromatography and MS alignment issues. Moreover, quantitative MS analysis of all individual samples is time consuming and requires a high-performance computer. Therefore, the pooled samples were used in the initial analysis to minimize these issues. Then, both fractions were analyzed separately by label-free quantitative proteomics. This was helpful for obtaining more peptides of low- and high-abundant proteins for MS analysis in terms of both identification and quantification. Based on our proteomics analysis, the levels of LRG, S100A8, C9, FN, AGP1, and A1AT were altered in the CRC group with the metastatic stage-dependent manner. These candidate proteins were confirmed by immunoblotting. Most results were consistent between two methods. This allowed us to gain more confidence in our study approach. In addition, the altered levels of these proteins determined in individual plasma samples of the study- and validation-cohorts demonstrated the possible uses of candidate biomarkers. As mentioned earlier, several blood-based proteins have been proposed as potential biomarkers of CRC; however, very few proteins could be applied to clinical practice [13–15]. In our study, except for the S100A8 level which varied in a wide range in each group, hampering the value of its biomarker into clinical application, other biomarker candidates demonstrated a metastatic stage-dependent manner to some extent.

LRG is a secreted protein involved in signal transduction, and cell adhesion and development. Its expression is up-regulated by the mediators of acute-phase response [16]. The roles of LRG in CRC were involved in promoting proliferation and inhibiting apoptosis [17] and promoting angiogenesis [18]. Consistent with our findings, LRG level was reported to be up-regulated in the plasma of CRC patients [19]. However, circulating LRG level is increased in many cancers and other diseases i.e. diabetes [20]. This suggests that LRG may not be a specific biomarker for any particular disease.

C9 is one member of the complement membrane attack complex (MAC) which normally plays a vital role in the immune response by forming pores in target cell membranes, causing cell lysis. However, growing evidence reported that its level was increased in the serum/plasma of patients with esophageal cancer [21] and gastric cancer [22]. Previously, our group also reported an elevated C9 level in the plasma of CRC patients detected by a different quantitative proteomics approach [10]. In the present study, the increased C9 level was detected in an independent cohort, suggesting that it would be a strong biomarker candidate for CRC.

FN is a high-molecular-weight glycoprotein which exists in a soluble form found in blood and in an insoluble form resides in the extracellular matrix of tissues. Due to the presence of many ligand-binding domains, resulting in the activation of various signaling pathways, it regulates cellular processes including cell adhesion, growth, migration, and differentiation [23]. Altered FN expression has been associated with many pathologies including cancer [24]. However, the roles of FN in tumor initiation and progression are highly controversial [24]. In this study and a previous report by our group [10], plasma FN level is significantly decreased in CRC patients with the metastatic stage-dependent manner. Consistent with this finding, Bogdanovic et al. showed that FN level was down-regulated in tissues and absent in the plasma of patients with CRC liver metastases [25]. Moreover, Zhou et al., reported that the serum FN level was decreased in patients with metastatic non-small cell lung cancer in comparison to those with non-metastatic lung cancer patients [26]. On the other hand, a number of reports suggested that FN level was up-regulated in many types of cancer [27–29]. The conclusions have been diverse, which may be due to the original source of FN and complicating conditions of patients such as inflammation. Further investigation is needed to seek the precise mechanism of FN in tumorigenesis, especially in CRC.

A1AT, also known as SERPINA3, is an acute-phase protein that has various biological roles including anti-inflammation, immunomodulatory, anti-infective and tissue-repair molecule [30]. Aberrant levels of blood A1AT have been reported in CRC [31] and many other cancers such as bladder cervical cancers [5, 32]. A1AT is now being adopted in a commercial bladder cancer diagnostic test kit, Oncuria™, where a panel of 10 biomarkers exhibited a great discriminatory power of 93% sensitivity and specificity [33]. In our study, we found that plasma A1AT level was up-regulated in CRC patients.

AGP1 is an acute-phase protein found in plasma. It is primarily produced in liver and peripheral tissues in response to systemic inflammation. AGP1 has been also reported as a potential biomarker for some cancers such as laryngeal cancer and pancreatic ductal adenocarcinoma [34, 35]. The up-regulation of serum AGP1 level was also associated to distant metastasis of patients with larynegeal cancer [34]. Consistent with these reports, we also found that plasma AGP1 level was up-regulated in CRC patients. Moreover, AGP1 level could be distinguished between two disease stages, suggesting that it may play roles in cancer progression.

### Combinations of biomarker candidates

Diagnostic performance of single and combined candidate proteins was analyzed in terms of sensitivity and specification. As shown in Fig. 4, FN exhibited the best diagnostic performance in discriminating between CRC patients and healthy controls in both study- and validation-cohorts. However, according to our data and others, a single biomarker candidate may not be applicable for CRC diagnosis. This may be because the single biomarker candidate, so far, was not specifically altered in only patients with cancer but also in other diseases. In addition, multiple pathological pathways may be involved during the initiation and progression of cancer which diminishes the significance of a single candidate biomarker. Therefore, biomarker panels represent a promising solution, as [1] they are involved in pathological tumor pathways and [2] the sensitivity and specificity of the combined tests can be greatly improved. In this study, the combination of 3 biomarker candidates, FN + A1AT + AGP1, conferred 100% sensitivity and specificity for CRC diagnosis in both cohorts. Compared to other efficient biomarker panels that may require up to 10 biomarkers, e.g. to identify bladder cancer [33] and steroid-resistant nephrotic syndrome [36], our biomarker panel has a considerable advantage in terms of production cost, which will be a great benefit to most people in reaching an efficient CRC screening test at an affordable price.

### Possible involvement of abnormal plasma glycoproteins in CRC patients

In terms of functional annotation, the possible involvement of proteins which their expressions were highly altered in NM-CRC and M-CRC groups was analyzed. Involvement in the classical complement activation (GO: 0006958) was revealed in both NM- and M-CRC groups, while the acute-phase response (GO: 0006953) and inflammatory response (GO:0006954) were linked only to the M-CRC group. The more GO found in the M-CRC group compared to those in the NM-CRC group may be considered as various functional pathways related to tumor progression.

Complement activation is an important process leading to inflammatory responses in both innate and adaptive immunity. Activation of the complement system generates anaphylatoxins (C3A and C5A) and membrane attack complex (C5B-9) and opsonizes targeted cells. The consequence of complement activation promotes cell dedifferentiation, proliferation, and migration [37]. Complement activation in the tumor microenvironment also enhances tumor growth and increases metastasis [37]. In this study, we found that the plasma levels of C4A, C4B, C1qC, and C9 were up-regulated in CRC patients. The elevation of these complement components may reflect the more active complement system in CRC patients, especially C9, which may provide indirect evidence of malignancy.

Acute-phase response is a consequence in response to inflammation which mainly results from the alteration of a group of acute-phase proteins (APPs) present in plasma blood. A report indicates that the levels of many APPs including A1AT were altered in patients with various cancers [38]. According to our proteomics analysis, we found that the levels of several APPs including LRG, A1AT, AGP1, FN, and HP were altered. In addition, an elevation of C-reactive protein (CRP), a predominant protein of the acute phase response, was also detected in the M-CRC patient group (in the supplementary data, Table S2 [sheet “MARS14_FT (no conflict)], only one peptide found and quantified). Elevated blood CRP levels have been used as an invasive index of any ongoing inflammatory response, however its increase is proposed to contribute to tumor progression through reactive oxygen species and cytokine signaling in the tumor microenvironment [39]. Thus, altered expression levels of these APPs in CRC patients would play vital roles not only in inflammatory responses but also in tumor development.

## Conclusions

Herein, we investigated the candidate biomarkers from plasma samples of patients with NM-CRC and M-CRC stages in comparison to those of healthy controls using the MARS-14 affinity chromatography and quantitative proteomics mass spectrometry analysis. Several plasma proteins were identified and quantified from both flow-through and elute fractions. Most of the proteins in which their expressions were highly altered in the patients with NM-CRC were mainly involved in complement activation, while those in those with M-CRC were clustered in acute-phase response, complement activation, and inflammatory response, and these pathways may contribute to cancer progression. Among them, the levels of LRG, C9, A1AT, and AGP1 were up-regulated while the level of FN were down-regulated with significantly statistical difference in CRC patients in comparison to those of healthy controls. In addition, we found that AGP1 could be discriminated between metastatic stages while it revealed a trend in up-regulation levels of LRG, C9, A1AT, and AGP1 while down-regulation level of FN in the metastatic stage-dependent manner. The combined use of 3 biomarker candidates, i.e. FN + A1AT + AGP1, improved the diagnostic performance over any single candidate in terms of CRC screening and disease stage discrimination. Altogether, we have expanded the spectrum of candidate biomarkers of CRC screening. However, since this study used a small sample size, validation our findings will require follow-up studies of the biomarker panels with a larger CRC population, as well as additional disease control groups and other types of cancer, to determine whether the differences in these biomarker candidates can be substantiated between CRC stages as well as in healthy controls. In addition, the use of rapid test such as ELISA should be applied for detecting these biomarkers.

## Electronic Supplementary Material

Below is the link to the electronic supplementary material

**Supplementary Table S1**: List of Plasma samples of patients with coloreactal cancer (CRC) and healthy controls and their clinical data used in the study-and validation-cohorts

**Supplementary Table S2**: Label-free Quantitative MS

**Supplementary Table S3**: Functional analysis of the differentially expressed plasma proteins in CRC patients

**Supplementary Table S4**: The expression levels of six proteins in individual plasma samples in the study cohort determined by westernblotting

**Supplementary Table S5**: The expression levels of six proteins in individual plasma samples in the validation cohort determined by westernblotting

**Supplementary Table S6**: ROC analysis of biomarker candidates and their combination to discriminate between CRC patients and healthy controls

**Supplementary Table S7**: ROC analysis of biomarker candidates and their combinations to discriminate between pateints with non-metastatic and metastatic CRC

## Data Availability

All data generated or analyzed in this study are included in this published article and its supplementary information files. The raw MS and label-free quantitative proteomics data have been deposited to ProteomeXchange consortium via the Proteomic Identifications (PRIDE) partner repository with the dataset identifier PXD040999 (40)

## List of Abbreviations

ADAMDEC1: A disintegrin and metalloproteinase domain-like protein decysin 1
ANOVA: Analysis of Variance
AGP1: Alpha-1-acid glycoprotein 1
APOC3: Apolipoprotein C-III
APPS: Acute-phase proteins
AUC: Area under the curve
A1AT: Alpha-1-antitrypsin
CA125: Cancer antigen 125
CA19-9: Carbohydrate antigen 19 − 9
CA72-4: Cancer antigen 72 − 4
CEA: Carcinoembryonic antigen
CFD: Complement factor D
COMP: Cartilage oligomeric matrix protein
CRC: Colorectal cancer
CRP: C-reactive protein
CST3: Cystatin C
C1qC: Complement C1q subcomponent subunit C
C4A: Complement C4-A
C4B: Complement C4-B
C9: Complement component C9
DAVID: Database for Annotation, Visualization and Integrated Discovery
EL: Elute
ELISA: Enzyme-linked immunosorbent assay
FDR: False discovery rate
FGF-21: Fibroblast growth factor 21
FN: Fibronectin
FT: Flow-through
GO: Gene Ontology
GPX3: Glutathione peroxidase 3
HBB: Hemoglobin subunit beta
HC: Healthy controls
HP: Haptoglobin
HPLC: High performance liquid chromatography
HSP90B1: Endoplasmin (Heat shock protein 90 kDa beta member)
LC-MS/MS: Liquid chromatography coupled with tandem mass spectrometry
IGHG4: Immunoglobulin heavy constant gamma 4
LRG: Leucine-rich alpha-2-glycoprotein-1
NM: Non-metastatic
M: Metastatic
MRC1: Mannose receptor C-type 1
MARS-14: Human 14 Multiple Affinity Removal System
PBP: Platelet basic protein
PON1: Paraoxonase 1
PPY: Pancreatic polypeptide
ROC: Receiver operating characteristic
S100A8: Protein S100-A8
